# Intravenous Inoculation of a Bat-Associated Rabies Virus Causes Lethal Encephalopathy in Mice through Invasion of the Brain via Neurosecretory Hypothalamic Fibers

**DOI:** 10.1371/journal.ppat.1000485

**Published:** 2009-06-19

**Authors:** Mirjam A. R. Preuss, Marie-Luise Faber, Gene S. Tan, Michael Bette, Bernhard Dietzschold, Eberhard Weihe, Matthias J. Schnell

**Affiliations:** 1 Institute of Anatomy and Cell Biology, Department of Molecular Neuroscience, Philipps University Marburg, Marburg, Germany; 2 Jefferson Vaccine Center, Department of Microbiology and Immunology, Thomas Jefferson University, Philadelphia, Pennsylvania, United States of America; University of North Carolina, United States of America

## Abstract

The majority of rabies virus (RV) infections are caused by bites or scratches from rabid carnivores or bats. Usually, RV utilizes the retrograde transport within the neuronal network to spread from the infection site to the central nervous system (CNS) where it replicates in neuronal somata and infects other neurons via trans-synaptic spread. We speculate that in addition to the neuronal transport of the virus, hematogenous spread from the site of infection directly to the brain after accidental spill over into the vascular system might represent an alternative way for RV to invade the CNS. So far, it is unknown whether hematogenous spread has any relevance in RV pathogenesis. To determine whether certain RV variants might have the capacity to invade the CNS from the periphery via hematogenous spread, we infected mice either intramuscularly (i.m.) or intravenously (i.v.) with the dog-associated RV DOG4 or the silver-haired bat-associated RV SB. In addition to monitoring the progression of clinical signs of rabies we used immunohistochemistry and quantitative reverse transcription polymerase chain reaction (qRT-PCR) to follow the spread of the virus from the infection site to the brain. In contrast to i.m. infection where both variants caused a lethal encephalopathy, only i.v. infection with SB resulted in the development of a lethal infection. While qRT-PCR did not reveal major differences in virus loads in spinal cord or brain at different times after i.m. or i.v. infection of SB, immunohistochemical analysis showed that only i.v. administered SB directly infected the forebrain. The earliest affected regions were those hypothalamic nuclei, which are connected by neurosecretory fibers to the circumventricular organs neurohypophysis and median eminence. Our data suggest that hematogenous spread of SB can lead to a fatal encephalopathy through direct retrograde invasion of the CNS at the neurovascular interface of the hypothalamus-hypophysis system. This alternative mode of virus spread has implications for the post exposure prophylaxis of rabies, particularly with silver-haired bat-associated RV.

## Author Summary

Rabies virus (RV) infects mammalian neurons and cycles in regionally distinct animal populations such as the red fox in Europe, domestic canines in Asia, or raccoons, skunks and bats in Northern America. Although human rabies can be prevented by pre- and post-exposure prophylaxis, more than 50,000 people die annually from the severe encephalopathy caused by RV. Recently, two cases of RV transmission by organ transplantation were reported. In our study, using intravenous inoculation of mice, we evaluated the pathogenetic relevance of virions that reach the bloodstream. Mice inoculated intravenously with a canine-derived RV survived the infection in contrast to intramuscularly injected mice, while mice infected with a silver-haired bat-related RV succumbed to the disease regardless of the route of inoculation. We found that the silver-haired bat-related RV was able to transit from the blood to the brain by invading neurosecretory fibers of the hypothalamus, which form neurohemal synapses lacking a blood-brain-barrier. This newly described route of brain invasion might reflect how RV reached the central nervous system from transplanted organs, since it takes longer to establish the neural connections between host and grafted tissue necessary for classical RV migration than the time until the infection became symptomatic in the two reported cases.

## Introduction

Rabies is a fatal central nervous system (CNS) disease in mammals, caused by rabies virus (RV), a neurotropic lyssavirus from the family of the rhabdoviridae [1]. Generally, RV is transmitted by scratches or bites of rabid animals, which results in the dissemination of virions into skin and muscle tissue. After initial infection of cells at the infection site, RV enters axon terminals and migrates by retrograde axonal transport into the CNS [2]–[4], where it causes a lethal encephalopathy. The incubation period can vary from days to years [5],[6]; however, it is not known where the virus resides during this time.

It is likely that a part of the virus that is introduced into damaged muscle or skin tissue after a bite is disseminated into the blood and transported via blood circuits to the CNS. Such an event could play a role in virus transmission by silver-haired bats where only few virus particles are minimally invasively introduced into small patches of skin, which have only few intraepidermal nerve fibers and therefore are not favorable for neuronal uptake [7],[8].

In contrast to natural RV infections, experimental RV infections are commonly done by intramuscular (i.m.), intranasal or intracerebral inoculation. Although injection into muscle probably imitates best natural infections, it causes much less local damage of skin, muscle tissue and microvasculature than an animal bite. Therefore, incidental hematogenous spread due to injury of vessels is less likely than in natural transmissions.

Our study aimed to elucidate the pathogenetic relevance of hematogenous RV spread. In particular, we wanted to examine whether RV is able to directly invade the brain from the vasculature and where this invasion would preferentially take place. To accomplish this, we infected mice i.m. or intravenously (i.v.) with the dog-associated RV strain DOG4 or the silver-haired bat associated RV strain SB. Both of these RV strains are highly neuroinvasive [9], but differ greatly in their neurotropism. While DOG4 infects almost exclusively neuronal cells, SB can readily infect other cells in vitro such as endothelial cells and fibroblasts [10].

## Results

### Intravenously Inoculated SB, but not DOG4, Is Highly Pathogenic

**Figure 1:**
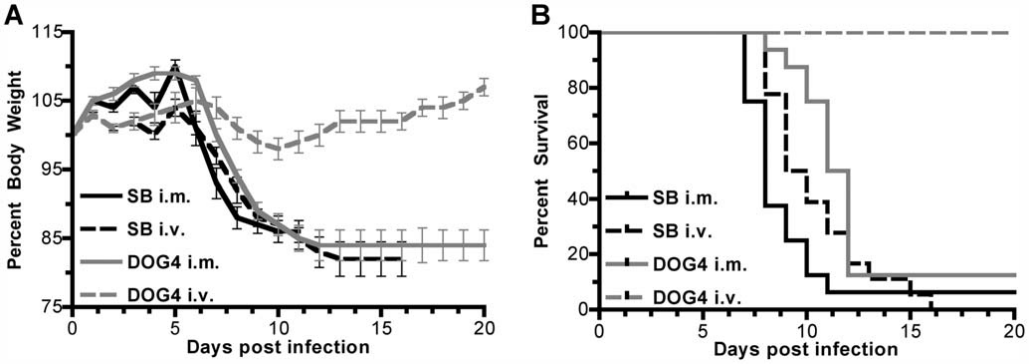
Strain and Inoculation Route Dependent Pathogenicity of SB and DOG4 in Mice. Six- to eight-week-old mice were inoculated i.m. or i.v. with 10^6^ ffu of SB or DOG4. (A) The graph depicts the weight curves of ten mice per group as group averages (mean±standard error). Percentages relate to the body weight at the day of inoculation. (B) Kaplan-Meier plots show the survival probability per day for each experimental group. Data are combined from two independent experiments. Survival curves were statistically different (p<0.0001) between SB i.m. (n = 16) and DOG4 i.m. (n = 17), SB i.v. (n = 18) and DOG4 i.v. (n = 17) as well as between DOG4 i.m. and DOG4 i.v.

As a first step to obtain evidence that certain RV variants might have the capacity to reach the brain from a peripheral site via hematogenous spread, we infected mice i.m. or i.v. with either DOG4 or SB. After i.m. injection of 10^6^ focus forming units (ffu) of SB (group 1) or DOG4 (group 2), all mice developed classical rabies symptoms like fur ruffling, weight loss (Figure 1A), hunchback posture and hind limb paralysis. 94% of the SB-infected and 88% the DOG4-infected mice succumbed to the infection with average survival times of 8.3±1.2 and 10.9±1.2 days, respectively. In contrast to the i.m. inoculation, only mice that were inoculated i.v. with SB (group 3) developed rabies with a mortality rate of 100%, while all mice which were inoculated i.v. with DOG4 (group 4) survived. The mean survival time after SB i.v. infection (10.4±2.4 days) was not significantly different from the survival time after SB i.m. infection (p>0.05, Figure 1B). Although the disease onset as indicated by the loss of body weight was alike in both SB groups (Figure 1A), mice infected i.v. with SB did not develop hind limb paralysis. Of note, i.v. injected SB caused disease symptoms similar to those reported for intracerebral inoculation [11].

### Distribution of RV Antigen in the CNS Differs after SB and DOG4 Inoculation

Since symptoms and outcome were different between the four experimental groups, the CNS of one mouse infected i.m. or i.v. with SB or DOG4 was analyzed by immunohistochemistry to get an overview of the viral distribution in the moribund stage (group 1–3) or at the end of the 20-day observation period, respectively (group 4).

**Figure 2:**
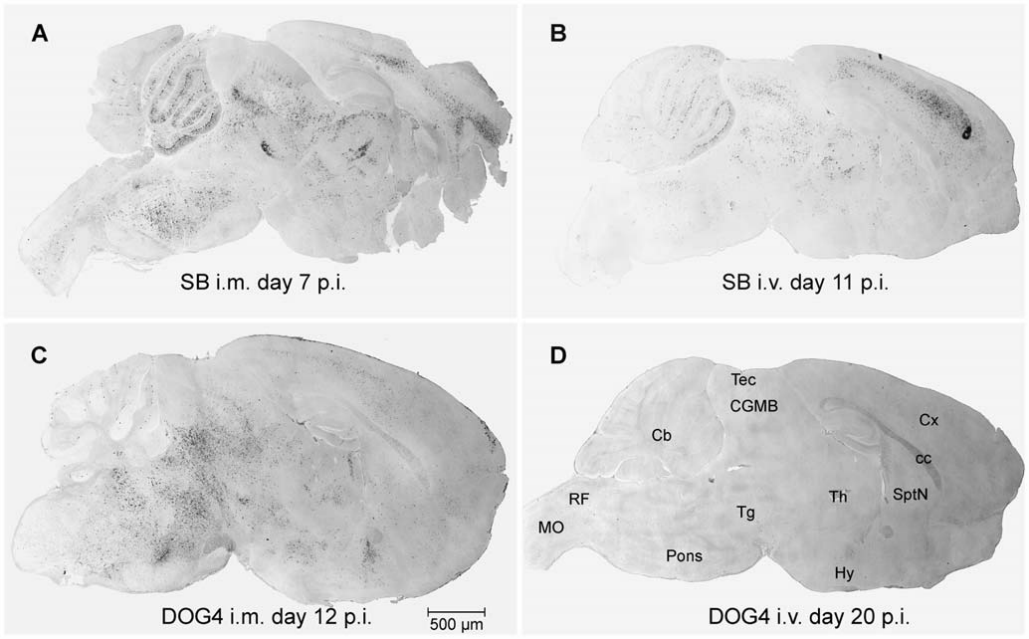
Strain and Inoculation Route Dependent Distribution of Viral Antigen in the Brain. Brains of mice inoculated i.m. or i.v. with 10^6^ ffu of SB or DOG4 were analyzed when the animals were moribund or at the end of the 20-day observation period as indicated in the panels A to D. Sagittal sections were stained immunohistochemically against RV, subsequently visualized by an enzymatic reaction and documented with bright field microscopy. Abbreviations: Cb, cerebellum; cc, corpus callosum; CGMB, central gray substance of midbrain; Cx, cerebral cortex; Hy, hypothalamus; MO, medulla oblongata; RF, reticular formation; SptN, septal nuclei; Tg, midbrain tegmentum; Tec, tectum; Th, thalamus.

Independent from the inoculation route, SB was present in the CNS of the two moribund animals in brainstem, cerebellum, thalamus and neocortex (Figure 2A and 2B). However, the virus load was more prominent in the central gray of midbrain and in neocortical areas after i.v. inoculation as compared to i.m. infection.

In contrast, DOG4 infected only neurons in the midbrain tegmentum and the brainstem when injected i.m., but not in the neocortex or the cerebellum (Figure 2C). In the brain of the i.v. DOG4 infected mouse, no viral antigen was detectable at all (Figure 2D). The spinal cord of this animal was also completely free of viral antigen in contrast to the spinal cord of the mice from the other three groups (data not shown).

**Figure 3:**
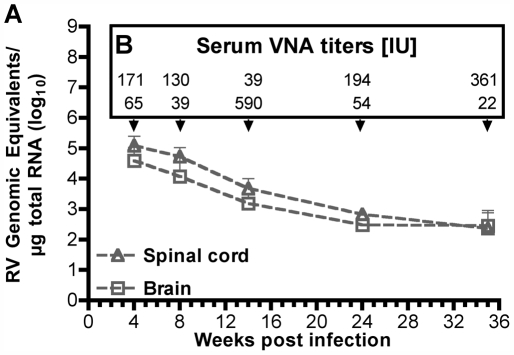
Residual DOG4 RNA in the CNS and Serum VNA Titers after i.v. Inoculation. Ten mice were infected i.v. with 10^6^ ffu of DOG4 and euthanized pair wise at the indicated time points. (A) The number of RV genome equivalents per microgram total RNA isolated from spinal cord and brain was quantified by qRT-PCR. Data are mean RNA copy numbers (+standard error) calculated for each mouse pair. (B) From the same mice, blood was obtained immediately before euthanasia and VNA serum titers were determined in international units (IU).

On the RNA level, DOG4 genomes were detectable by quantitative reverse transcriptase polymerase chain reaction (qRT-PCR) over a course of at least eight months at low quantities, decreasing from 10^5^ to 5×10^2^ copies per microgram total RNA (Figure 3A). Despite the presence of viral RNA, isolation of infectious virions from two brains 35 weeks post infection failed, which might be explainable by the very high virus neutralizing antibody (VNA) serum titers that were produced in all animals (Figure 3B). These results, together with the transient weight loss suggest that at least a transient infection of CNS tissue after DOG4 i.v. inoculation did occur.

### Progression of SB Invasion into the CNS is Different after Intravenous and Intramuscular Inoculation

**Figure 4:**
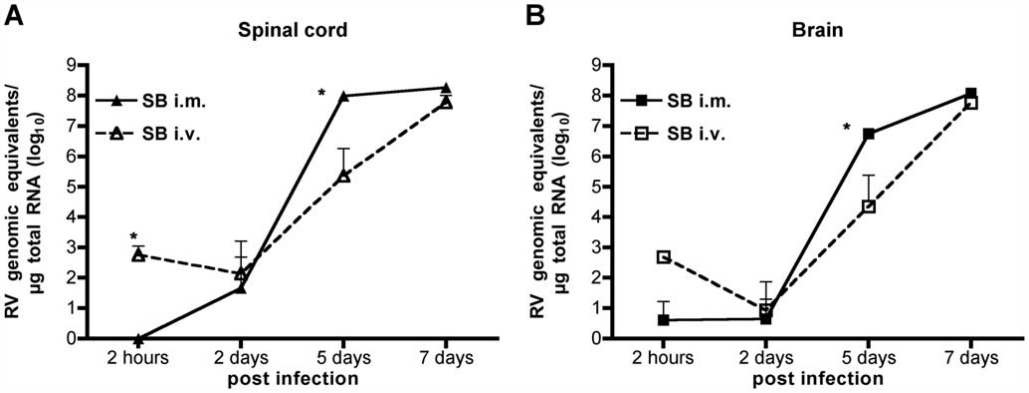
Viral Burden in the CNS after SB i.m. or i.v. Inoculation. Twelve mice were infected i.m. or i.v. with 5×10^6^ ffu of SB. Three mice per group were euthanized at each indicated time point and the number of RV genome equivalents per microgram total RNA isolated from spinal cord (A) and brain (B) was quantified by qRT-PCR. Data are mean RNA copy numbers (+standard error) calculated for three mice per time point. Asterisks indicate significant differences in the virus load at the indicated time point in dependence on the inoculation route (*, p<0.05).

In order to analyze the migration pathways after i.v. and i.m. SB infection, the virus load in brain and spinal cord was monitored by qRT-PCR and immunohistochemical analysis. RNA from spinal cord and brain tissue was harvested from mice early after inoculation (2 hours post infection), before the onset of symptoms (2 days post infection), at the beginning of weight loss (5 days post infection) and in the progressed stage of disease (7 days post infection) and analyzed for SB genomic equivalents (Figure 4A and 4B). After i.m. inoculation, genomic SB RNA was detectable in the spinal cord in fairly low amounts only at day 2 post infection, but the virus load rapidly increased over more than six logs within the following three days (Figure 4A). In the brain, genomic SB RNA was detectable in higher concentrations only at day 5 post infection, but in very low amounts also at day 2 and even already 2 hours post infection (Figure 4B). The latter finding supported our working hypothesis that spill over of virus into the vascular system and spread throughout the whole organism can occur after i.m. inoculation. In comparison, after i.v. inoculation SB RNA was detectable in significantly (spinal cord, p<0.05) or slightly (brain) higher amounts especially at the early time points, whereas the amplification slope from the day of infection to day 7 post infection was significantly steeper in the CNS of i.m. inoculated mice (p<0.05); this became particularly noticeable at day 5 post infection in both CNS segments.

**Figure 5:**
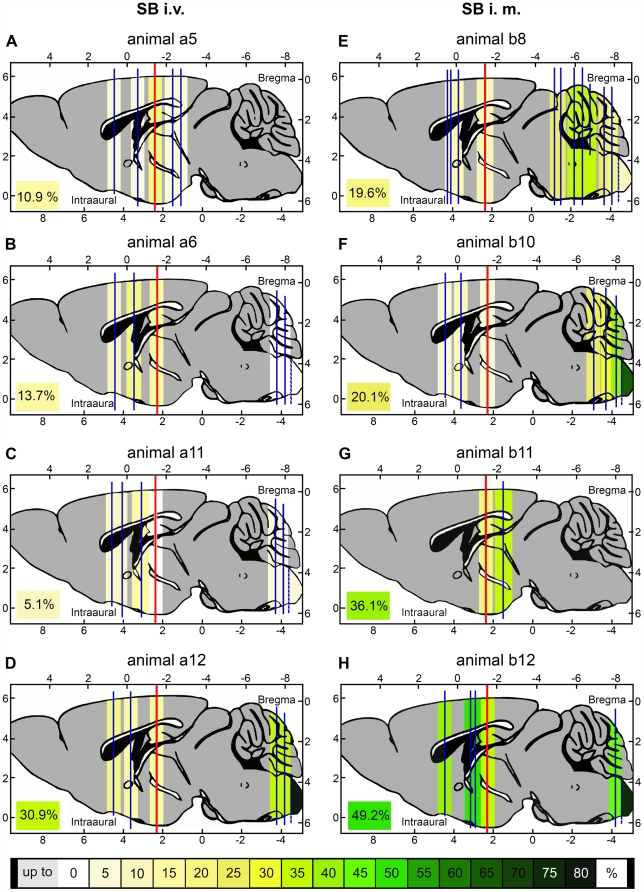
SB Progression within the CNS after i.m. or i.v. Inoculation. CNS tissue from four of twelve mice infected either i.v. (panel A–D) or i.m. (panel E–H) with 10^7^ ffu of SB was analyzed in more detail by immunohistochemical staining against RV. Animal numbers relate to Table 1. The red bar represents the section plane (stereotaxic coordinates: interaural 2.34 mm, bregma −1.46 mm) for the initial screening of all mouse brains (see text). Each vertical blue bar stands for another plane that was analyzed by several sections. The area caudal of the broken blue line represents the findings for the cervical spinal cord. The background color of each plane symbolizes its percentage of SB positive structures (nuclei, fiber tracts, cortical areas). Distinct structures were identified and counted using a stereotaxic mouse brain atlas [43]. Gray colored regions were not analyzed. The total percentage of RV immunoreactive structures for each brain is noted in the left corner of each panel.

The similar results obtained for the two inoculation modes regarding the extent of viral burden in the late stages of the disease indicated that difference in clinical symptoms between the groups is not due to the virus load in the CNS but to a difference in spatial localization within the CNS, especially in the earlier stages of the disease. In order to test this, the virus load in RV prone regions such as the hippocampus, thalamic and hypothalamic nuclei, basal ganglia, the amygdala, as well as primary and secondary somatosensory cortex was compared by immunohistochemical stainings against RV (Table 1; Figure 5, red bar). In the i.m. infected mice, viral antigen was only found when an animal exhibited first motor deficits, assessed by its performance in the trunk curl test (morbidity status ++), and vice versa (Table 1). In contrast, brains of i.v. infected animals did not reveal any correlation between the presence of virus in the screened plane and the morbidity status (Table 1).

**Table 1:** Morbidity and Virus Load at Different Time Points after i.m. or i.v. Inoculation of SB.

SB i.v.				SB i.m.			
Days post infection	Animal	Morbidity^1^	Virus load^2^	Days post infection	Animal	Morbidity	Virus load
2	a1	−	−	2	b1	−	−
	a2	−	−		b2	−	−
	a3	−	−		b3	−	−
	a4	−	−		b4	−	−
4	*a5*	*−*	*++*	4	b5	−	−
	*a6*	*−*	*++*		b6	+	−
	a7	+	−		b7	+	−
	a8	+	−		*b8*	*++*	*++*
7	a9	++	−	6	b9	++	+
	a10	++	−		*b10*	*++*	*+*
	*a11*	*+++*	*−*		*b11*	*++*	*+++*
	*a12*	*++++*	*++*		*b12*	*+++*	*++++*

Twelve mice were inoculated with 10^7^ ffu of SB either by i.v. or i.m. injection and monitored for symptoms until mice were euthanized in a still healthy state (2 days post infection), at the onset of weight loss (4 days post infection) and in the progressed symptomatic stage (7 days after i.v. respectively 6 days after i.m. infection). Virus load was assessed for all animals by immunohistochemistry against RV in matched frontal brain sections (stereotaxic coordinates: interaural 2.34 mm, bregma −1.4 mm). Animals in italic typeset were chosen for further analysis.

1 From ‘−’, no signs of illness, to ‘+++++’, morbid.

2 From ‘−’, no immunoreactivity against RV RNP, to ‘++++’, many RV RNP immunoreactive neurons and fibers in several areas.

Four of twelve animals were chosen from each group (Table 1, italic typeset) for a more detailed immunohistochemical study of on average more than 80 CNS structures. The color-coding in Figure 5 visualizes a spatiotemporal, wavelike progression of SB from the spinal cord and brainstem to the forebrain after i.m. inoculation (Figure 5E–5H). In contrast, infection of spinal cord or brainstem was not a prerequisite for infection of higher-order structures after i.v. inoculation (Figure 5A).

### For SB, Neurosecretory Fibers of the Neurohypophysis and the Median Eminence Are Access Points from the Vascular System to the Forebrain

**Figure 6:**
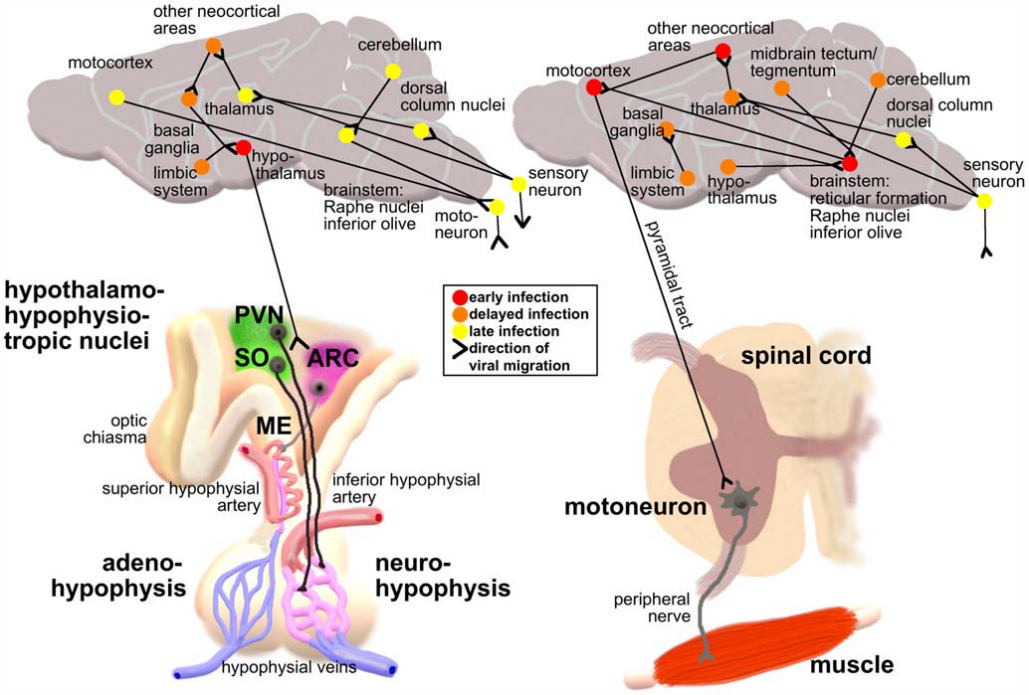
Potential Viral Invasion and Migration Pathways after i.v. (left) or i.m. (right) SB Inoculation. Potential viral migration pathways between all RV antigen positive cortical areas and nuclei in brain and spinal cord, analyzed and identified as described in Figure 5, were composed manually by using the BrainInfo database (http://braininfo.rprc.washington.edu/) for published efferent and afferent connections. Depicted connections were chosen by evaluation of all possibilities with regard to the best match for each animal as well as between the animals in dependence on time and morbidity. Presented is a simplified version, summarized on the level of hierarchically higher-ordered structures.

In order to retrace viral invasion into and spread within the CNS all immunohistochemically stained sections were further analyzed on the level of defined structures (nuclei, cortical areas, fiber tracts) and their retrograde connections among each other (Figure 6). This revealed motoneurons in the ventral horn of the spinal cord to be the exclusive source for the further spread of SB into the brain after i.m. inoculation (Figure 6, right). Following i.m. inoculation, SB reached the motocortex via the pyramidal tract, from whence it migrated to premotocortical and primary somatosensory areas as well as motor-related brainstem nuclei (inferior olive, reticular formation, raphe nuclei). Originating from the olive, virus spread to the deep nuclei and the cortex of the cerebellum. Via the reticular formation, the basal ganglia became affected, and through the raphe nuclei, SB reached hypothalamic nuclei as well as structures in the midbrain. During progression of the disease, various thalamic nuclei, further hypothalamic areas, limbic structures and other neocortical regions got infected. Only then was virus also found in sensory-related thalamic areas (ventral posterior complex), brainstem nuclei (dorsal column nuclei) and spinal cord laminae in the dorsal horn.

**Figure 7:**
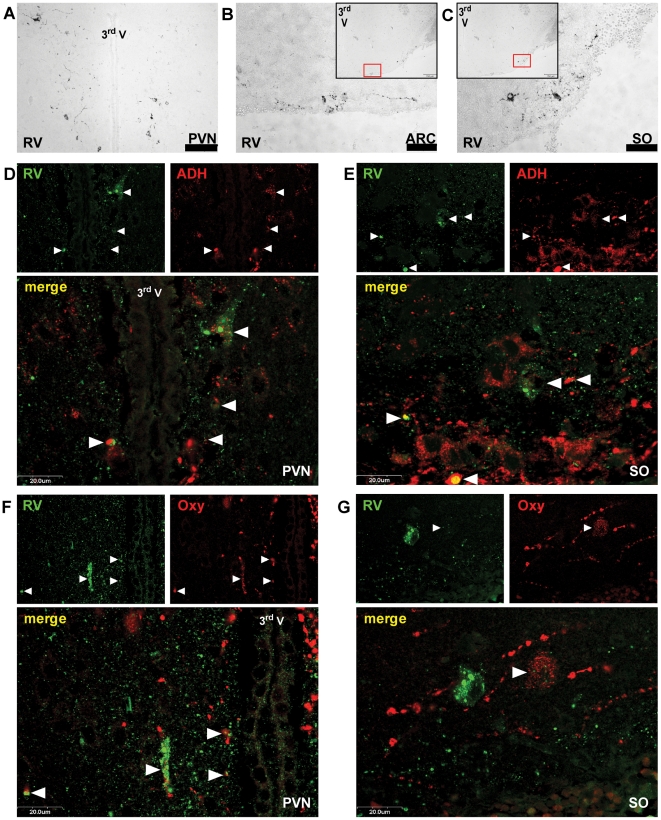
Infection of Neurosecretory Hypothalamic Neurons by SB after i.v. Inoculation. The panels show the hypothalamic paraventricular (PVN; panel A, D and F), arcuate (ARC; panel B) and supraoptic (SO; panel C, E and G) nuclei of mice inoculated i.v. with 10^7^ SB four days before euthanasia. The sections were stained immunohistochemically against RV only (panel A–C) or co-stained against RV and antidiuretic hormone (ADH; panel D and E) or oxytocin (Oxy; panel F and G). Abbreviation: 3^rd^ V, third ventricle. Black or white bars represent 20 (panel D–G), 25 (panel B, C) or 75 µm (panel A). Arrowheads point to neurons and fibers containing both RV and ADH, or Oxy respectively.

At four days after i.v. inoculation of SB (the earliest time point, at which viral antigen could be detected immunohistochemically), the only SB positive structures were the hypothalamic paraventricular (PVN; Figure 7A) and supraoptic (SO; Figure 7C) nuclei, which send neurosecretory axons to the neurohypophysis (NPH), as well as the arcuate (ARC) nucleus (Figure 7B), which is connected to the median eminence (ME). ME and NPH are secretory circumventricular organs (CVO), where the usually tight blood-brain-barrier is discontinued by fenestrated capillaries in order to allow the release of neuron-derived hormones at neurohemal synapses. Immunofluorescent co-stainings revealed that SB resided in oxytocin- (Figure 7F and 7G) as well as in antidiuretic hormone-positive (Figure 7D and 7E) fibers of the SO and PVN. Other structures to which SB also spread while still in the asymptomatic phase (further hypothalamic areas; basal ganglia: striatum, globus pallidus; other telencephalic nuclei: amygdala, nucleus accumbens, septal nuclei) were all accessible to the virus via their efferents to the affected hypothalamic nuclei. In the advanced stage of the disease, however, the same structures were infected than after i.m. inoculation suggesting a late second invasion of the CNS on conventional pathways independent from the early infection of the basal forebrain (Figure 6, left).

## Discussion

We show here that two street RV strains that are equally virulent in mice after i.m. infection differ completely in their pathogenicity when they are inoculated into the bloodstream. While i.v. inoculation of DOG4 did not cause any clinical signs of rabies, i.v. infection with SB resulted in a lethal encephalopathy. However, the early transient weight loss together with the detection of viral RNA in the CNS and the development of high VNA serum titers indicate that an infection of the brain did occur after i.v. inoculation of DOG4 but that it had apparently no or only little consequences on the health of the animals. The presence of RV in apparently healthy animals has been reported before [12],[13] It is not known, however, if these animals were only presymptomatic when they were examined, or if it the infection was under control. The failure to detect viral antigen immunohistochemically in CNS tissue of DOG4 i.v. infected mice in spite of the presence of viral RNA can be explained by the different sensitivities of RT-qPCR and the histochemical analysis. In addition, the attempt to isolate virus from the brains of two i.v. DOG4 infected mice 35 weeks pi likely failed because of the presence of VNA in the tissue homogenates [14]. Similar observations were previously made with the neurotropic Sindbis virus. This pathogen with a positive sense single-stranded RNA genome causes acute encephalitis in mice, but is usually cleared within eight days: virus isolation from brain failed as soon as 8 days pi [15], viral protein was not detectable by immunohistochemistry later than two weeks and RNA by in situ hybridization later than 20 days pi [16],[17] while demonstration of viral RNA by RT-PCR was still successful 24 weeks after infection [18]. Antibodies constitute the main effectors against RV [19],[20]. They are able to prevent rabies when given in the presymptomatic stage [21], or to clear apathogenic strains in experimental infections [22]. A conclusive explanation for the low and decreasing rate of virus production we observed in the CNS of DOG4 i.v. infected mice could be the inhibition of virus production by antibodies, a defense mechanism that is more beneficial for the host than the killing of virus-infected neurons [17], [19], [23]–[25]. A possible reactivation of the DOG4 virus in the CNS and development of a lethal encephalopathy when anti-RV antibody levels have decreased cannot be excluded, as shown for Sindbis virus [26]. Future experiments will have to further characterize the immune and infection status of DOG4 i.v. infected mice in order to clarify if the prolonged presence of DOG4 RNA in the CNS has any pathological relevance.

The strain-specific differences in the distribution of viral antigen in the CNS suggest that the disparity in the survivorship of mice after i.v. inoculation of DOG4 or SB is likely due to differences in their neurotropism [27],[28]. However, they also indicate that the symptoms in the progressed stages of the disease, which were similar after i.m. infection with SB or DOG4 but different after i.v. infection with SB, are not determined by the infection of particular regions of the brain. The finding that SB was able to directly invade neurosecretory fibers from the vascular system, while i.v. injected DOG4 failed to establish a lethal infection of the brain by using this way of entry, suggests that both viruses may use different cell surface molecules that facilitate their uptake. However, since dog- as well as bat-associated RV strains can vary substantially in their neurotropism [9], it is possible that different bat RV strains might actually utilize different cellular attachment molecules like the neural cell adhesion molecule, the neurotrophin receptor p75TNR, distinct subunits of the nicotinic acetylcholine receptor or other yet undefined molecules, which could vary with the specific glycoproteins of these viruses. Therefore, future experiments must reveal whether our findings can be generalized for other canine- and bat-associates RV strains.

The migration of SB can be precisely traced after i.m. inoculation. Immunohistochemical analysis shows that motoneurons in the spinal cord are the exclusive source for virus progression into higher-order CNS structures; this is in agreement with a previous study made in skunks [29]. The fact that early infection can only be detected in the ventral horn neurons excludes the possibility that SB uses sensory fibers in addition to motor axons for CNS invasion from the i.m. inoculation site as it has been proposed for other RV strains [30]–[32].

The observed symptoms of mice after i.v. inoculation strongly suggest that SB directly infects the brain [11]. Our conclusion that SB invades the CNS at the neurovascular interface of the hypothalamus-hypophysis system, is somewhat in conflict with an earlier report [33] that the transit of virus into neuronal tissue occurs via infection of ependymal cells that line the ventricles which contain the cerebral liquor transsudated from vessels of the choroid plexus. In addition, the possibility of passing the blood-brain-barrier via nicotinic acetylcholine receptor-mediated endocytosis into endothelial cells of brain capillaries [34], as it has been suggested in a recent study [35], can be excluded by our findings. A third possibility is the invasion of SB via CVO, which are highly vascularized sites that facilitate direct communication of neurons with blood and liquor through fenestrated endothelium. CVO either consist of neuronal cell bodies and sense various circulating substances (sensory CVO), or they are formed by neurosecretory axons and glial cells (secretory CVO). Their special composition exposes them also as targets for invasion of pathogens, such as trypanosoma [36]. The retrograde invasion of SB from vessels into the CNS through neurosecretory fibers of the CVO ME and NPH is strongly indicated, because mice sacrificed four days after i.v. inoculation showed an almost exclusive involvement of the hypothalamic nuclei PVN, SO and ARC. PVN and SO project oxytocin- and antidiuretic hormon-secreting axons to the NPH, and ARC is considered to form a functional complex together with the ME [37], where hormones for the regulation of the adenohypophysis are released.

In summary, our study revealed a route of brain invasion alternative to the migration in peripheral nerves, which might be advantageous for certain RV strains. The transit from the vascular system to the brain via neurosecretory fibers of the ME and NPH might also present a possible explanation for the RV infection of immunosuppressed recipients of RV infected organs that was reported in 2004 in the U.S.A. and in 2005 in Germany [38],[39]. Transplanted tissue is deprived of direct nerval input for many months [40]; thus the classical retrograde pathway of CNS invasion by RV via visceromotor fibers of the autonomic nervous system would have still been unavailable at the time of the actual incubation for the reported transplantation incidents. Instead, virions present in the transferred organs might have been spilled into the recipients' bloodstream and reached the CNS on this newly described route.

## Materials and Methods

### Viruses

The SB strain was rescued from a cDNA clone that was derived from the SHBRV 18 strain [28] and passaged on BSR cells. The DOG4 strain was originally isolated from a human brain and passaged on mouse neuroblastoma cells [9].

### Mouse experiments

Six- to eight-week-old female Swiss Webster mice were commercially obtained (NCI-Frederick Animal Production Area, http://web.ncifcrf.gov/; Taconic Farms, http://www.taconic.com/) and used for all in vivo studies. For i.m. infections virus-containing Dulbecco's phosphate buffered saline was injected into the right gastrocnemius muscle. Intravenous inoculation was performed by injecting virus into the heat-dilated tail vein. The injured vein was treated with a cautery device to minimize viral spread into the surrounding tissue by closing off the needle puncture and severing nearby axons and nerve terminals through which virus could be taken up from the inoculation site. Mice were monitored and scored daily for weight changes and signs of rabies.

All animal experiments were performed according to Institutional Animal Care and Use Committee-approved protocols (Animal Welfare Assurance no. A3085-01).

### Tissue harvest

Brains and spinal cord for RNA isolation were immersed into an appropriate amount (1 ml per 100 mg tissue) of RNAlater RNA Stabilization reagent (Qiagen, www.qiagen.com/) immediately after harvest from the euthanized mouse and stored at 4°C for maximal four weeks until further processing.

Brains and spinal cords determined for immunohistochemical analysis were immersion-fixed in Bouin Hollande fixative for 24 hours and washed with 70% isopropanol afterwards.

Brains from which infectious particles were intended to be isolated were put promptly onto ice and processed within few hours.

Murine blood was drawn by heart puncture under isoflurane anesthesia before euthanizing the animal. To prevent coagulation, syringe and collection tube were flushed with 8% ethylenediamine tetraacetic acid. Blood for serological tests was kept on ice until centrifugation for ten minutes at 16,000×g. The serum was kept at 4°C until analysis.

### Immunohistochemical analysis

Immunohistochemical stainings of CNS tissue was performed as previously described [41].

For enzymatic immunostainings that were done to trace viral migration pathways in the CNS, tissue sections were incubated with a polyclonal rabbit antibody raised against the RV ribonucleoprotein complex (RNP), diluted 1∶3,000 (source: B. Dietzschold).

Confocal double-immunofluorescence stainings were performed to identify SB target cells in the hypothalamus-hypophysis-system. Brain sections were incubated with a combination of the polyclonal mouse antibody Rabies NMAS 802-3 raised against RV RNP (source: B. Dietzschold), diluted 1∶300, and one of the following antibodies: (i) rabbit-anti-oxytocin, 1∶300 (ICN Biomedicals, Frankfurt am Main, Germany); (ii) rabbit-anti-antidiuretic hormone, 1∶500 (ICN Biomedicals).

### Quantification of RV genome equivalents by qRT-PCR

#### RNA purification

Total RNA from solid tissue (brains and spinal cords) was isolated using a handheld homogenizer with disposable probes (Omni International, www.omni-inc.com/) for disruption and the RNeasy Mini kit (Qiagen) according to the manufacturer's instructions.

A 15-minute on-column DNase I digest (Qiagen) was included for all samples during the purification procedure.

#### Primer and TaqMan probe design

All primers and probes for the qRT-PCR were designed by the program Primer3 (http://frodo.wi.mit.edu/cgi-bin/primer3/primer3_www.cgi) using as input the leader and RV N nucleotide sequence of the RV strains SHBRV18 (Entrez Gene accession number: AY705373), which is identical to that of SB, and DOG4.

#### Two-step qRT-PCR

One microgram of total RNA was reverse-transcribed by using Omniscript reverse transcriptase (Qiagen) according to the manufacturer's protocol and a gene-specific primer for genomic RV RNA (RP381, 5′- ACACCCCTACAATGGATGC-3′). The reactions were incubated for 1 hour at 37°C and the enzyme was subsequently inactivated by 5 minutes at 95°C. Quantitative PCR reactions were set up in LightCycler capillaries (Roche Applied Science, https://www.roche-applied-science.com/). Each 20 µl-reaction contained 1× QuantiTect Probe PCR Master Mix (Qiagen), 500 nM forward primer (RP381), 500 nM reverse primer (RP382 for DOG4, 5′-GGGTTATACAGGGCTTTTTCA-3′; RP405 for SB, 5′-ATTCATGCCAGACAAAATTGA-3′), 100 nM TaqMan probe (RTP-3 for SB, 5′-FAM-TACAAGTACCCGGCAATCAAAGACTCG-TAMRA-3′; RTP-5 for DOG4, 5′-FAM-CAATAATCAGGTGGTCTCTTTGAAGCCAGA-TAMRA-3′) and 100 ng of template cDNA. Reactions were performed as triplicates in a LightCycler 1.5 instrument (Roche Applied Science) along with a positive control triplicate and a no-template control by using the following cycling protocol: hot start 15 minutes at 95°C; amplification 45 cycles [15 seconds at 95°C, 60 seconds at 60°C]; cooling 30 seconds at 40°C. The fluorescence of the hydrolyzed probes was measured in a single step at the end of each amplification cycle, and threshold cycles were obtained by the second derivative method through the LightCycler software v.3.5.3 (Roche Applied Science). For absolute quantification, a standard curve was generated for SB and DOG4 genomic RNA from serial dilutions of cDNA of a known copy number (SB: y = −3.3773x + 42.282, R^2^ = 0.9998, 98% efficiency; DOG4: y = −3.3224x + 40.973, R^2^ = 0.9999, 100% efficiency) and the copy numbers were normalized to 1 µg/µl total RNA.

### VNA assay

Sera were tested for the presence of VNA using the rapid fluorescence inhibition test [42]. The neutralizing titer, defined as the inverse of the highest serum dilution that neutralizes 50% of the challenge virus, was normalized to IU using the World Health Organization anti-RV antibody standard.

### Virus isolation from tissue

Brains intended for virus isolation were weighed, and sterile Dulbecco's phosphate buffered saline was added to get a 20%-organ suspension after disruption with a handheld homogenizer and disposable probes (Omni International). The homogenates were centrifuged for 10 minutes at 1,000×g. Subsequently, 100 µl cleared homogenate were added to a cell pellet of 7.5×10^7^ murine neuroblastoma cells, together with 10 µg DEAE-dextran and 100 µl serum-free Roswell Park Memorial Institute (RPMI) 1640 medium with L-glutamine. After 30 minutes at 37°C, the mixture was spun down for 3 minutes at 3,000×g, and the pellet was re-suspended in 1 ml 5% fetal bovine serum-containing RPMI 1640. The cells were seeded in T25 flasks, and the volume was raised to 10 ml. After two days at 37°C, cells were stained with a FITC-conjugated anti-RV N-antibody (Fujirebio Diagnostics, www.fdi.com/) according to the manufacturer's recommendations and analyzed for the presence of viral antigen.

### Statistical analysis

Kaplan-Meier survival curves were analyzed by the log rank test. qRT-PCR data were analyzed by two-way ANOVA and Bonferroni post tests to determine if differences in the virus load of a tissue at the indicated time point depended on the inoculation route. All data were analyzed using GraphPad Prism 4.0 software (GraphPad Software, www.graphpad.com/).

## References

[1] Pringle CR (1991). The order Mononegavirales.. Arch Virol.

[2] Bulenga G, Heaney T (1978). Post-exposure local treatment of mice infected with rabies with two axonal flow inhibitors, colchicine and vinblastine.. J Gen Virol.

[3] Tang Y, Rampin O, Giuliano F, Ugolini G (1999). Spinal and brain circuits to motoneurons of the bulbospongiosus muscle: retrograde transneuronal tracing with rabies virus.. J Comp Neurol.

[4] Kelly RM, Strick PL (2000). Rabies as a transneuronal tracer of circuits in the central nervous system.. J Neurosci Methods.

[5] Smith JS, Fishbein DB, Rupprecht CE, Clark K (1991). Unexplained rabies in three immigrants in the United States. A virologic investigation.. N Engl J Med.

[6] McColl KA, Gould AR, Selleck PW, Hooper PT, Westbury HA (1993). Polymerase chain reaction and other laboratory techniques in the diagnosis of long incubation rabies in Australia.. Aust Vet J.

[7] Gibbons RV (2002). Cryptogenic rabies, bats, and the question of aerosol transmission.. Ann Emerg Med.

[8] Hemachudha T, Laothamatas J, Rupprecht CE (2002). Human rabies: a disease of complex neuropathogenetic mechanisms and diagnostic challenges.. Lancet Neurol.

[9] Dietzschold B, Morimoto K, Hooper DC, Smith JS, Rupprecht CE (2000). Genotypic and phenotypic diversity of rabies virus variants involved in human rabies: implications for postexposure prophylaxis.. J Hum Virol.

[10] Morimoto K, Patel M, Corisdeo S, Hooper DC, Fu ZF (1996). Characterization of a unique variant of bat rabies virus responsible for newly emerging human cases in North America.. Proc Natl Acad Sci U S A.

[11] Johnson RT (1965). Experimental rabies. Studies of cellular vulnerability and pathogenesis using fluorescent antibody staining.. J Neuropathol Exp Neurol.

[12] Aghomo HO, Rupprecht CE (1990). Further studies on rabies virus isolated from healthy dogs in Nigeria.. Vet Microbiol.

[13] Fekadu M, Shaddock JH, Chandler FW, Baer GM (1983). Rabies virus in the tonsils of a carrier dog.. Arch Virol.

[14] Iwasaki Y, Gerhard W, Clark HF (1977). Role of host immune response in the development of either encephalitic or paralytic disease after experimental rabies infection in mice.. Infect Immun.

[15] Griffin DE, Johnson RT (1977). Role of the immune response in recovery from Sindbis virus encephalitis in mice.. J Immunol.

[16] Jackson AC, Moench TR, Griffin DE, Johnson RT (1987). The pathogenesis of spinal cord involvement in the encephalomyelitis of mice caused by neuroadapted Sindbis virus infection.. Laboratory investigation; a journal of technical methods and pathology.

[17] Levine B, Hardwick JM, Trapp BD, Crawford TO, Bollinger RC (1991). Antibody-mediated clearance of alphavirus infection from neurons.. Science.

[18] Tyor WR, Wesselingh S, Levine B, Griffin DE (1992). Long term intraparenchymal Ig secretion after acute viral encephalitis in mice.. J Immunol.

[19] Dietzschold B, Kao M, Zheng YM, Chen ZY, Maul G (1992). Delineation of putative mechanisms involved in antibody-mediated clearance of rabies virus from the central nervous system.. Proc Natl Acad Sci USA.

[20] Hooper DC, Morimoto K, Bette M, Weihe E, Koprowski H (1998). Collaboration of antibody and inflammation in clearance of rabies virus from the central nervous system.. J Virol.

[21] WHO (2005). WHO Expert Consultation on rabies.. World Health Organization technical report series.

[22] Hooper DC, Morimoto K, Bette M, Weihe E, Koprowski H (1998). Collaboration of antibody and inflammation in clearance of rabies virus from the central nervous system.. J Virol.

[23] Alexandersen S, Larsen S, Cohn A, Uttenthal A, Race RE (1989). Passive transfer of antiviral antibodies restricts replication of Aleutian mink disease parvovirus in vivo.. J Virol.

[24] Fujinami RS, Oldstone MB (1979). Antiviral antibody reacting on the plasma membrane alters measles virus expression inside the cell.. Nature.

[25] Fujinami RS, Oldstone MBA (1980). Alterations in expression of measles virus polypeptides by antibody: molecular events in antibody-induced antigenic modulation.. J Immunol.

[26] Levine B, Griffin DE (1992). Persistence of viral RNA in mouse brains after recovery from acute alphavirus encephalitis.. J Virol.

[27] Morimoto K, Foley HD, McGettigan JP, Schnell MJ, Dietzschold B (2000). Reinvestigation of the role of the rabies virus glycoprotein in viral pathogenesis using a reverse genetics approach.. J Neurovirol.

[28] Faber M, Pulmanausahakul R, Nagao K, Prosniak M, Rice AB (2004). Identification of viral genomic elements responsible for rabies virus neuroinvasiveness.. Proc Natl Acad Sci U S A.

[29] Charlton KM, Casey GA, Wandeler AI, Nadin-Davis S (1996). Early events in rabies virus infection of the central nervous system in skunks (Mephitis mephitis).. Acta Neuropathol.

[30] Harrison AK, Murphy FA (1978). Lyssavirus infection of muscle spindles and motor end plates in striated muscle of hamsters.. Arch Virol.

[31] Lycke E, Tsiang H (1987). Rabies virus infection of cultured rat sensory neurons.. J Virol.

[32] Coulon P, Derbin C, Kucera P, Lafay F, Préhaud C (1989). Invasion of the peripheral nervous systems of adult mice by the CVS strain of rabies virus and its avirulent derivative AvO1.. J Virol.

[33] Jackson AC, Reimer DL (1989). Pathogenesis of experimental rabies in mice: an immunohistochemical study.. Acta Neuropathol.

[34] Gotti C, Clementi F (2004). Neuronal nicotinic receptors: from structure to pathology.. Prog Neurobiol.

[35] Kumar P, Wu H, McBride JL, Jung KE, Kim MH (2007). Transvascular delivery of small interfering RNA to the central nervous system.. Nature.

[36] Schultzberg M, Ambatsis M, Samuelsson EB, Kristensson K, van Meirvenne N (1988). Spread of Trypanosoma brucei to the nervous system: early attack on circumventricular organs and sensory ganglia.. J Neurosci Res.

[37] Yi CX, van der Vliet J, Dai J, Yin G, Ru L (2006). Ventromedial arcuate nucleus communicates peripheral metabolic information to the suprachiasmatic nucleus.. Endocrinology.

[38] Hellenbrand W, Meyer C, Rasch G, Steffens I, Ammon A (2005). Cases of rabies in Germany following organ transplantation.. Euro Surveill.

[39] Burton EC, Burns DK, Opatowsky MJ, El-Feky WH, Fischbach B (2005). Rabies encephalomyelitis: clinical, neuroradiological, and pathological findings in 4 transplant recipients.. Arch Neurol.

[40] Arrowood JA, Minisi AJ, Goudreau E, Davis AB, King AL (1997). Absence of parasympathetic control of heart rate after human orthotopic cardiac transplantation.. Circulation.

[41] Faber M, Bette M, Preuss MAR, Pulmanausahakul R, Rehnelt J (2005). Overexpression of tumor necrosis factor alpha by a recombinant rabies virus attenuates replication in neurons and prevents lethal infection in mice.. J Virol.

[42] Wiktor TJ, Macfarlan RI, Foggin CM, Koprowski H (1984). Antigenic analysis of rabies and Mokola virus from Zimbabwe using monoclonal antibodies.. Dev Biol Stand.

[43] Paxinos G, Franklin KB (2001). The mouse brain in stereotaxic coordinates, 2^nd^ edition.

